# Methotrexate-related adverse events and impact of concomitant treatment with folic acid and tumor necrosis factor-alpha inhibitors: An assessment using the FDA adverse event reporting system

**DOI:** 10.3389/fphar.2023.1030832

**Published:** 2023-02-22

**Authors:** Kenji Onda, Takeshi Honma, Koichi Masuyama

**Affiliations:** ^1^ Department of Clinical Pharmacology, School of Pharmacy, Tokyo University of Pharmacy and Life Sciences, Tokyo, Japan; ^2^ Bohsei Pharmacy, Kanagawa, Japan; ^3^ Regulatory Science laboratory, School of Pharmacy, Tokyo University of Pharmacy and Life Sciences, Tokyo, Japan

**Keywords:** methotrexate, FAERS database, folic acid, TNFalpha inhibitor, interstitial lung disease, hepatotoxicity, myelosuppresion, tuberculosis

## Abstract

Methotrexate (MTX) is an essential anti-rheumatic drug used to treat rheumatoid arthritis (RA). Prevention or management of adverse reactions, including interstitial lung disease (ILD), hepatotoxicity, myelosuppression, and infection, remains fundamental for safe MTX therapy. Using the Food and Drug Administration (FDA) Adverse Event Reporting System (FAERS) (JAPIC AERS), we performed disproportionality analyses of adverse events related to MTX use and the impact of concomitant medications. Upon analyzing all reported cases in FAERS between 1997 and 2019, the crude reporting odds ratios (cRORs; 95% confidence intervals) for ILD, hepatotoxicity, myelosuppression, and tuberculosis (TB) in relation to MTX use were 4.00 (3.83–4.17), 1.99 (1.96–2.02), 3.66 (3.58–3.74), and 7.97 (7.65–8.3), respectively. Combining MTX with folic acid (FA) or tumor necrosis factor-alpha inhibitors (TNFis) tended to reduce cRORs for these adverse events (except for TB). Multiple logistic regression analysis in patients with RA was conducted to calculate adjusted reporting odds ratios (aRORs) for age, sex, and MTX treatment patterns (MTX alone and combined with FA and TNFi). Higher age (except for hepatotoxicity) and male sex were significantly associated with adverse events. Combining FA or TNFi with MTX reduced aRORs for MTX-related hepatotoxicity and myelosuppression; in contrast, the effect of FA was not obvious in ILD or TB. Although studies assessing spontaneous reporting systems have limitations such as reporting bias, data from our logistic regression analysis demonstrated that adding FA to MTX-based therapy could help reduce the dose-dependent adverse events of MTX, thereby providing clinical evidence that supports the beneficial effect of FA. This study also demonstrated the usefulness of FAERS in comparing adverse events based on treatment patterns.

## 1 Introduction

Methotrexate (MTX) is used as an anchor drug to treat rheumatoid arthritis (RA). Although highly efficacious, adverse reactions such as interstitial lung disease (ILD), hepatotoxicity, myelosuppression, and infections occasionally limit MTX use, and constant vigilance and management of these side effects are required to ensure safe MTX therapy.

ILD is a serious, life-threatening adverse reaction. The incidence of MTX-induced ILD ranges from 0.3% to 11.6% (Atzeni et al., 2013; Fragoulis et al., 2019). Early prevention and detection are crucial for successful MTX therapy. ILD was not associated with MTX dosage or duration of therapy (Jakubovic et al., 2013). Moreover, mechanisms related to allergic reactions have been suggested, although poorly understood. In contrast, hepatotoxicity and gastrointestinal symptoms were found to increase with increasing MTX dose. Bone marrow suppression is another dose-dependent adverse reaction associated with MTX therapy. These adverse events are associated with the MTX-induced deficiency of folic acid (FA), an important factor in cell proliferation.

Reportedly, the concomitant use of FA can effectively prevent dose-dependent adverse events, including hepatotoxicity, mucositis, and gastrointestinal symptoms. The addition of FA to MTX therapy has also been associated with a reduced frequency of MTX discontinuation (Shea et al., 2014). However, the preventive effect of FA on ILD is yet to be established (Jakubovic et al., 2013), while the preventive effect of FA on myelosuppression warrants further validation (Shea et al., 2013).

Infection is another side effect of MTX therapy attributed to its immunosuppressive effects. A recent meta-analysis has reported that MTX is associated with an increased risk of infection in patients with RA (Ibrahim et al., 2018). Furthermore, the use of tumor necrosis factor-alpha (TNFα) inhibitors (TNFis) increases the risk of infection, including tuberculosis (TB) and hepatitis B virus. Combined use of TNFi and MTX was found to increase the risk of infection (Greenberg et al., 2010; Fleischmann et al., 2017). Therefore, patients undergoing MTX and TNFi therapy require continuous and careful monitoring before and during treatment.

Treatment of RA focuses on MTX therapy combined with TNFi and other medications, such as classical and biological disease-modifying anti-rheumatic drugs (DMARDs). Although the combination of therapeutic agents may influence the risk of certain adverse events, limited comparative information is available on how trends in the occurrence of each adverse event differ for individual treatment patterns, including MTX. Such information is critical for safe MTX therapy tailored to the patient’s background.

The Food and Drug Administration (FDA) Adverse Event Reporting System (FAERS), a spontaneously reported adverse event database maintained by the FDA, collects information regarding all types of adverse drug events (Sakaeda et al., 2013). In particular, large databases such as FAERS are useful for analyzing infrequent adverse drug reactions. Disproportionality analysis is used to detect signals of adverse drug events (Bate and Evans, 2009). In addition, the application of FAERS has been proposed for analyzing drug interactions or identifying drugs that mitigate side effects (Gandhi et al., 2013; Zhao et al., 2013; Iyer et al., 2014; Nagaoka et al., 2021). Given the nature of spontaneous reporting data (for example, the true frequency of adverse drug reactions cannot be aggregated or the presence of reporting bias), directly comparing values of reporting odds ratios (RORs) obtained by disproportionality analysis can be deemed inappropriate. However, multivariate analysis with logistic analysis has been proposed to compare adjusted RORs (aRORs) under certain conditions (Suzuki et al., 2015; Oshima et al., 2018).

In the present study, we used the FAERS database to explore two major perspectives. First, cases of MTX use were selected from among all FAERS reported cases, and crude RORs (cRORs) of ILD, hepatotoxicity, myelosuppression, and TB as typical adverse drug reactions of MTX, were tabulated. Furthermore, cRORs of MTX combined with various drugs used to treat RA were calculated using univariate analysis to explore potential concomitant medications that could impact the cRORs of MTX-related adverse events. Second, a multivariate analysis was performed to calculate aRORs with age, sex, and pattern of concomitant medication use, which affected cRORs (FA or TNFi) as explanatory variables in patients with RA who used MTX. Based on these analyses, we attempted to determine whether different patterns of concomitant medications in MTX-containing therapy could affect the occurrence of MTX-related adverse events.

## 2 Materials and methods

### 2.1 Data source and mining

We used the JAPAN Pharmaceutical Information Center (JAPIC) FAERS data for all medications, inputted into FAERS between 1997 (4Q) and 2019 (1Q). This dataset was curated by deleting duplicate reports and proofing drug names. As FAERS is an anonymous public database, approval from the institutional review board was waived. The FAERS database contains several data tables. We used the DEMO (age, sex), DRUG (drug), REAC (adverse events), and INDI (indication) tables. Each table was connected to the primary ID and analyzed using the relational database software (Microsoft Access 2016).

### 2.2 Definition of adverse events

The adverse events were described using terms approved in the preferred term (PT) list from the Medical Dictionary for Regulatory Activities (MedDRA) ver. 22.0. ILD was defined using PT code 10022611 (Matsumoto et al., 2020). Hepatotoxicity was defined using the SMQ code 20000006 (Ikemura et al., 2019). Myelosuppression was defined using the SMQ code 20000028. TB was defined as the presence of 49 types of PTs related to TB. Complete lists of PT terms are listed in Supplementary Table S1.

### 2.3 Disproportionality analysis

**FIGURE 1:**
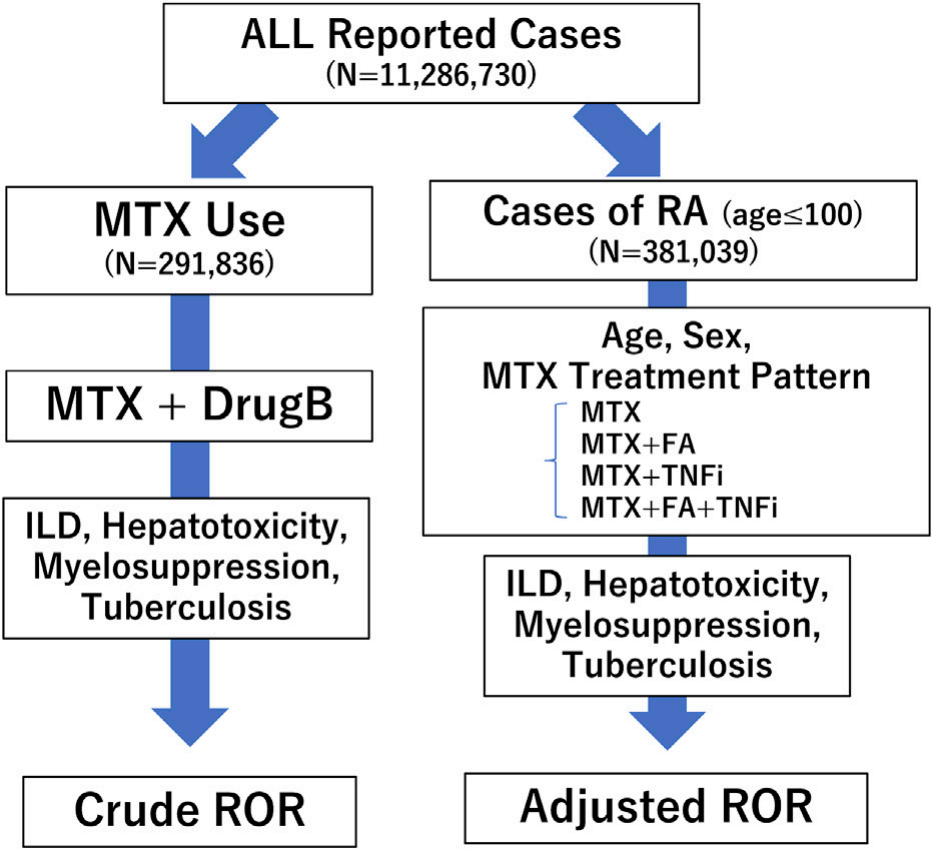
Flow chart for determining crude reporting odds ratios (cRORs) and adjusted RORs (aRORs) of methotrexate (MTX)-related adverse events. A disproportionality analysis was performed focusing on MTX use and respective adverse events such as interstitial lung disease (ILD), hepatotoxicity, myelosuppression, and tuberculosis (TB), considering all reported cases. Next, we selected patients with rheumatoid arthritis (RA) who were ≤100 years old and conducted a multiple logistic regression analysis to calculate aRORs for the adverse events. We set each adverse event as the objective variable and age, sex, and treatment patterns of MTX as explanatory variables. RORs, reporting odds ratios; MTX, methotrexate; ILD, interstitial lung disease; RA, rheumatoid arthritis; FA, folic acid; TNFi, tumor necrosis factor inhibitor.

**FIGURE 2:**
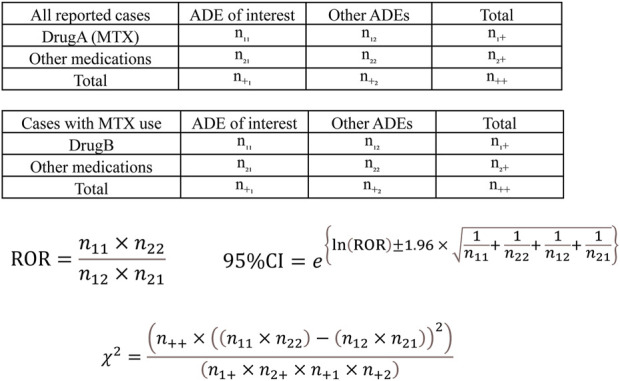
Calculation of the crude reporting odds ratios (cRORs), 95% confidence intervals (95% CIs), and χ^2^ values for each adverse drug event based on 2 × 2 contingency tables. cRORs for MTX (Drug A)-related adverse events were calculated with the upper 2 × 2 contingency table among all reported cases. The cRORs with concomitant drugs (Drug B) were calculated with the lower 2 × 2 contingency table among cases with MTX (Drug A) use. ADE, adverse drug event; MTX, methotrexate.

Analysis was performed according to the flowchart shown in Figure 1. We first conducted a disproportionality analysis by focusing on MTX use and respective adverse events (ILD, hepatotoxicity, myelosuppression, and TB), considering all reported cases. The analysis was based on the calculation of the number of cases in which a drug of interest (MTX as Drug A) was used and/or an adverse event of interest was reported (Figure 2). The cRORs, 95% confidence interval (CIs), and chi-squared (χ2) values were also calculated. Subsequently, we focused on cases that used MTX and evaluated cRORs for other concomitant medications (Drug B) used for RA therapy and each adverse event. Drugs were considered concomitantly used if listed in the same report as the primary ID.

### 2.4 Multiple logistic regression analysis for cases of RA

Next, we selected patients with RA who were ≤100 years old as judged from the INDI and DEMO tables and conducted a multiple logistic regression analysis to calculate aRORs for the adverse events. After excluding cases with unknown sex, we set each adverse event as the objective variable and age, sex, and treatment patterns of MTX as explanatory variables. We defined four treatment patterns of MTX: i) MTX group that did not use FA or TNFi, ii) MTX + FA group that did not use TNFi, iii) MTX + TNFi group that did not use FA, and iv) MTX + FA + TNFi group. TNFi was used if at least one TNFis (infliximab, adalimumab, etanercept, golimumab, or certolizumab) was employed. In our preliminary analysis to establish a logistic model, we confirmed that higher variance inflation factor (VIF) values were obtained with a logistic model incorporating the use of MTX, FA, and TNFi as covariables and factors of drug combination expressed as products (e.g., MTX*FA or MTX*FA*TNFi). Thus, we used an alternative model for logistic analysis as follows:

$$\mathrm{Log}\;\left(RORs\right)=\beta_{0}+\beta_{1}A+\beta_{2}S+\beta_{3}M_{1}+\beta_{4}M_{2}+\beta_{5}M_{3}+\beta_{6}M_{4}$$

$$(A=age,S=sex,M_{1}=MTX\;\left(no\;FA,TNFi\;\right),M_{2}=MTX+FA\;\left(no\;TNFi\right),M_{3}=MTX+TNFi\;\left(no\;FA\right),M_{4}=MTX+FA+TNFi)$$

Using this logistic model, we confirmed that all VIF values were ˂ 1.4, and the deviance value was statistically significant, supporting the model’s suitability.

Statistical significance was determined if the upper 95% CI of the ROR was ˂ 1.0 or the lower 95% CI of the ROR was ˃1.0. Fisher’s exact test was used to calculate the *p*-values of cRORs. Data mining and all statistical analyses were performed using Microsoft Access 2016 (Microsoft Inc. Tokyo, Japan), R version 3.4.1 (R Foundation for Statistical Computing, Vienna, Austria), EZR version 1.36 (Kanda, 2013), and GraphPad Prism ver. 9.2 (GraphPad Software, San Diego, CA).

## 3 Results

### 3.1 cRORs of MTX-related adverse events among all reported cases

In total, 11,286,730 cases were reported in the data-cleaned FAERS database. Of these, 291,836 were treated with MTX. The disproportionality analysis of MTX use and ILD, hepatotoxicity, myelosuppression, and TB resulted in cROR values (95% CI) of 4.00 (3.83–4.17), 1.99 (1.96–2.02), 3.66 (3.58–3.74), and 7.97 (7.65–8.3), respectively, all of which were significantly elevated (Table 1).

**TABLE 1:** Crude RORs (95% CI) for MTX-related adverse events in cases of MTX use.

Adverse event	With methotrexate			Without methotrexate			Crude ROR (95% CI)
	Cases	Non-cases		Cases	Non-cases		
	n_11_	n_12_	%	n_21_	n_22_	%	
Interstitial lung disease	2,366	289,470	0.81	22,415	10,972,479	0.20	4.00 (3.83–4.17)
Hepatotoxicity	20,111	271,725	6.89	394,776	10,600,118	3.59	1.99 (1.96–2.02)
Myelosuppression	8,322	283,514	2.85	87,496	10,907,398	0.80	3.66 (3.58–3.74)
Tuberculosis	2,821	289,015	0.97	13,452	10,981,442	0.12	7.97 (7.65–8.3)

CI, confidence interval; MTX, methotrexate; RORs, reporting odds ratios.

We then selected MTX use cases and calculated cRORs for MTX-related adverse events associated with the use of other concomitant drugs for treating RA. Table 2 presents data for TNFi and FA. Other concomitant drugs used for RA are listed in Supplementary Table S2. Concomitant TNFis, including adalimumab [0.76 (0.68–0.84)] and etanercept [0.9 (0.82–0.99)], and FA [0.89 (0.81–0.98)] resulted in significantly low cRORs for ILD (Table 2). Considering most other classical DMARDs, RORs remained high when combined with MTX (Supplementary Table S2). Similarly, in the case of hepatotoxicity, adalimumab, etanercept, certolizumab, golimumab, and FA presented significantly low cRORs, with values of 0.74 (0.72–0.77), 0.76 (0.73–0.78), 0.88 (0.81–0.96), 0.9 (0.82–0.98), and 0.52 (0.5–0.54), respectively (Table 2). Several other anti-rheumatic drugs could maintain high RORs, similar to those of ILD. In addition, on analyzing myelosuppression, adalimumab, infliximab, etanercept, certolizumab, golimumab, and FA exhibited statistically reduced RORs, with values of 0.13 (0.12–0.15), 0.26 (0.23–0.29), 0.16 (0.14–0.17), 0.14 (0.1–0.19), 0.19 (0.1–0.20), and 0.45 (0.43–0.48), respectively (Table 2). Considering other anti-rheumatic drugs, biological DMARDs, such as abatacept and tocilizumab, exhibited low RORs, whereas others presented high RORs (Supplementary Table S2). Furthermore, on analyzing TB, infliximab, certolizumab, golimumab, and FA presented significantly high RORs, with values of 5.78 (5.36–6.24), 2.12 (1.83–2.46), 1.39 (1.16–1.67), and 1.11 (1.02–1.2), respectively, whereas adalimumab and etanercept exhibited low RORs, with values of 0.83 (0.76–0.91) and 0.5 (0.45–0.55), respectively.

**TABLE 2:** Crude RORs (95% CI) for MTX–related adverse events in cases that used MTX and concomitant drugs.

Concomitant drugs	Interstitial lung disease		Hepatotoxicity		Myelosuppression		Tuberculosis	
Adalimumab	0.76 (0.68–0.84)	*l*	0.74 (0.72–0.77)	*l*	0.13 (0.12–0.15)	*l*	0.83 (0.76–0.91)	*l*
Infliximab	1.27 (1.12–1.42)	*h*	1.06 (1.01–1.1)	*h*	0.26 (0.23–0.29)	*l*	5.78 (5.36–6.24)	*h*
Etanercept	0.9 (0.82–0.99)	*l*	0.76 (0.73–0.78)	*l*	0.16 (0.14–0.17)	*l*	0.5 (0.45–0.55)	*l*
Certolizumab	1.99 (1.68–2.35)	*h*	0.88 (0.81–0.96)	*l*	0.14 (0.1–0.19)	*l*	2.12 (1.83–2.46)	*h*
Golimumab	1.78 (1.49–2.13)	*h*	0.9 (0.82–0.98)	*l*	0.19 (0.15–0.25)	*l*	1.39 (1.16–1.67)	*h*
Folic acid	0.89 (0.81–0.98)	*l*	0.52 (0.5–0.54)	*l*	0.45 (0.43–0.48)	*l*	1.11 (1.02–1.2)	*h*

*l* and *h* indicate RORs, were significantly low or high.

CI, confidence interval; MTX, methotrexate; RORs, reporting odds ratios.

### 3.2 Multiple regression analysis of MTX-related adverse events among patients with RA

Next, we stratified patients with RA to examine the impact of concomitant medications, particularly FA and TNFi, on MTX-related adverse events. We focused on FA and TNFi, given that they strongly tend to affect cRORs of adverse events. Cases of RA were extracted based on data in INDI tables, and cases that were ≤100 years with known sex were analyzed. Subsequently, patients with RA receiving MTX therapy were classified into four groups based on data regarding the use or non-use of FA or TNFi, as described in the Materials and Methods section. Using multiple logistic regression analysis, age, sex, and treatment patterns of MTX were set as explanatory variables, with each adverse event set as an objective variable.

Accordingly, RORs for the four adverse events, i.e., ILD, hepatotoxicity, myelosuppression, and TB, were all associated with older age (except hepatotoxicity, which was slightly associated with younger age) and male sex (Table 3). Overall, aRORs of the four MTX treatment patterns, except for the MTX + FA group in TB, showed significantly elevated aRORs.

**TABLE 3:** Crude and adjusted RORs (95% CI) for MTX-related adverse events calculated using the logistic regression analysis for each treatment pattern containing MTX in RA cases.

ILD								
Variables		Non-cases	Cases of ILD	Proportion of ILD (%)	Crude ROR	*p*-Value	Adjusted ROR	*p*-Value
Age, mean (SD)		58.22 (13.42)	65.56 (11.35)				1.04 (1.04–1.05)	<0.001
Sex	M	73,060	669	0.91 (0.84–0.98)	1.9 (1.73–2.09)	<0.001	1.71 (1.56–1.87)	<0.001
	F	303,982	1,463	0.48 (0.46–0.5)				
MTX		22,144	370	1.64 (1.48–1.82)	3.36 (3–3.77)	<0.001	4.73 (4.17–5.35)	<0.001
MTX + FA		9,601	167	1.71 (1.46–1.99)	3.25 (2.76–3.81)	<0.001	4.91 (4.15–5.8)	<0.001
MTX + TNFi		46,795	571	1.21 (1.11–1.31)	2.58 (2.34–2.84)	<0.001	4.03 (3.62–4.48)	<0.001
MTX + FA + TNFi		24,422	200	0.81 (0.7–0.93)	1.49 (1.28–1.73)	<0.001	2.7 (2.31–3.15)	<0.001

**Hepatotoxicity**								
Variables		Non-cases	Cases of hepatotoxicity	Proportion of hepatotoxicity (%)	Crude ROR	*p*-value	Adjusted ROR	*p*-value
Age, mean (SD)		58.26 (13.44)	58.22 (12.62)				0.997 (0.995–0.998)	<0.001
Sex	M	71,252	2,477	3.36 (3.23–3.49)	1.1 (1.05–1.15)	<0.001	1.07 (1.02–1.12)	0.005
	F	296,084	9,361	3.07 (3.01–3.13)				
MTX		20,619	1,895	8.42 (8.06–8.79)	3.2 (3.04–3.37)	<0.001	4.53 (4.29–4.78)	<0.001
MTX + FA		9,173	595	6.09 (5.63–6.58)	2.06 (1.89–2.25)	<0.001	3.17 (2.9–3.46)	<0.001
MTX + TNFi		44,490	2,876	6.07 (5.86–6.29)	2.32 (2.22–2.43)	<0.001	3.16 (3.02–3.31)	<0.001
MTX + FA + TNFi		23,647	975	3.96 (3.72–4.21)	1.3 (1.22–1.39)	<0.001	2.02 (1.88–2.16)	<0.001

**Myelosuppression**								
Variables		Non-cases	Cases of myelosuppression	Proportion of myelosuppression (%)	Crude ROR	*p*-value	Adjusted ROR	*p*-value
Age, mean (SD)		58.19 (13.40)	67.23 (12.56)				1.05 (1.05–1.05)	<0.001
Sex	M	72,981	748	1.02 (0.94–1.09)	1.45 (1.33–1.58)	<0.001	1.22 (1.12–1.33)	<0.001
	F	303,302	2,143	0.7 (0.67–0.73)				
MTX		21,366	1,148	5.1 (4.82–5.39)	10.73 (9.95–11.58)	<0.001	14.5 (13.3–15.9)	<0.001
MTX + FA		9,360	408	4.18 (3.79–4.59)	6.36 (5.71–7.08)	<0.001	11.5 (10.2–13)	<0.001
MTX + TNFi		46,979	387	0.82 (0.74–0.9)	1.07 (0.96–1.19)	0.216	2.61 (2.31–2.94)	<0.001
MTX + FA + TNFi		24,503	119	0.48 (0.4–0.58)	0.61 (0.5–0.73)	<0.001	1.56 (1.28–1.89)	<0.001

**Tuberculosis**								
Variables		Non-cases	Cases of tuberculosis	Proportion of tuberculosis (%)	Crude ROR	*p*-value	Adjusted ROR	*p*-value
Age, mean (SD)		58.25 (13.42)	59.53 (13.40)				1.01 (1–1.01)	<0.001
Sex	M	72,981	680	0.92 (0.86–0.99)	1.38 (1.27–1.51)	<0.001	1.36 (1.24–1.48)	<0.001
	F	303,404	2,041	0.67 (0.64–0.7)				
MTX		21,366	171	0.76 (0.65–0.88)	1.05 (0.9–1.23)	0.491	1.55 (1.32–1.82)	<0.001
MTX + FA		9,360	57	0.58 (0.44–0.76)	0.8 (0.62–1.04)	0.102	1.19 (0.91–1.55)	0.206
MTX + TNFi		46,979	802	1.69 (1.58–1.81)	2.93 (2.69–3.18)	<0.001	3.57 (3.26–3.9)	<0.001
MTX + FA + TNFi		24,503	394	1.6 (1.45–1.77)	2.44 (2.19–2.72)	<0.001	3.39 (3.03–3.8)	<0.001

CI, confidence interval; FA, folic acid; ILD, interstitial lung disease; MTX, methotrexate; ROR, reporting odds ratio; SD, standard deviation; TNFi, tumor necrosis factor-alpha inhibitor.

Considering ILD, the MTX group presented an aROR value of 4.73 (4.17–5.35), which was comparable with that of the MTX + FA group [4.91 (4.15–5.8)]. The aROR value of the MTX + TNFi group [4.03 (3.62–4.48)] was less than that of the MTX group. The MTX + FA + TNF group showed the smallest aROR of 2.7 (2.31–3.15).

Considering hepatotoxicity, younger age and male sex were weakly associated with aROR values. The aROR value of the MTX group [4.53 (4.29–4.78)] was the highest among the four treatment patterns. The aRORs of the MTX + FA [3.17 (2.9–3.46)] and MTX + TNFi [3.16 (3.02–3.31)] groups were comparable and less than that of the MTX group. The MTX + FA + TNFi combination resulted in the smallest aROR value [2.02 (1.88–2.16)]

Considering myelosuppression, the MTX group presented an aROR of 14.5 (13.3–15.9), the highest among the four treatment patterns, whereas the MTX + FA group exhibited a lower value [11.5 (10.2–13)]. The aROR of the MTX + TNFi group was reduced considerably [2.61 (2.31–2.94)], with the MTX + FA + TNFi group exhibiting a further reduction in the aROR value [1.56 (1.28–1.89)].

**FIGURE 3:**
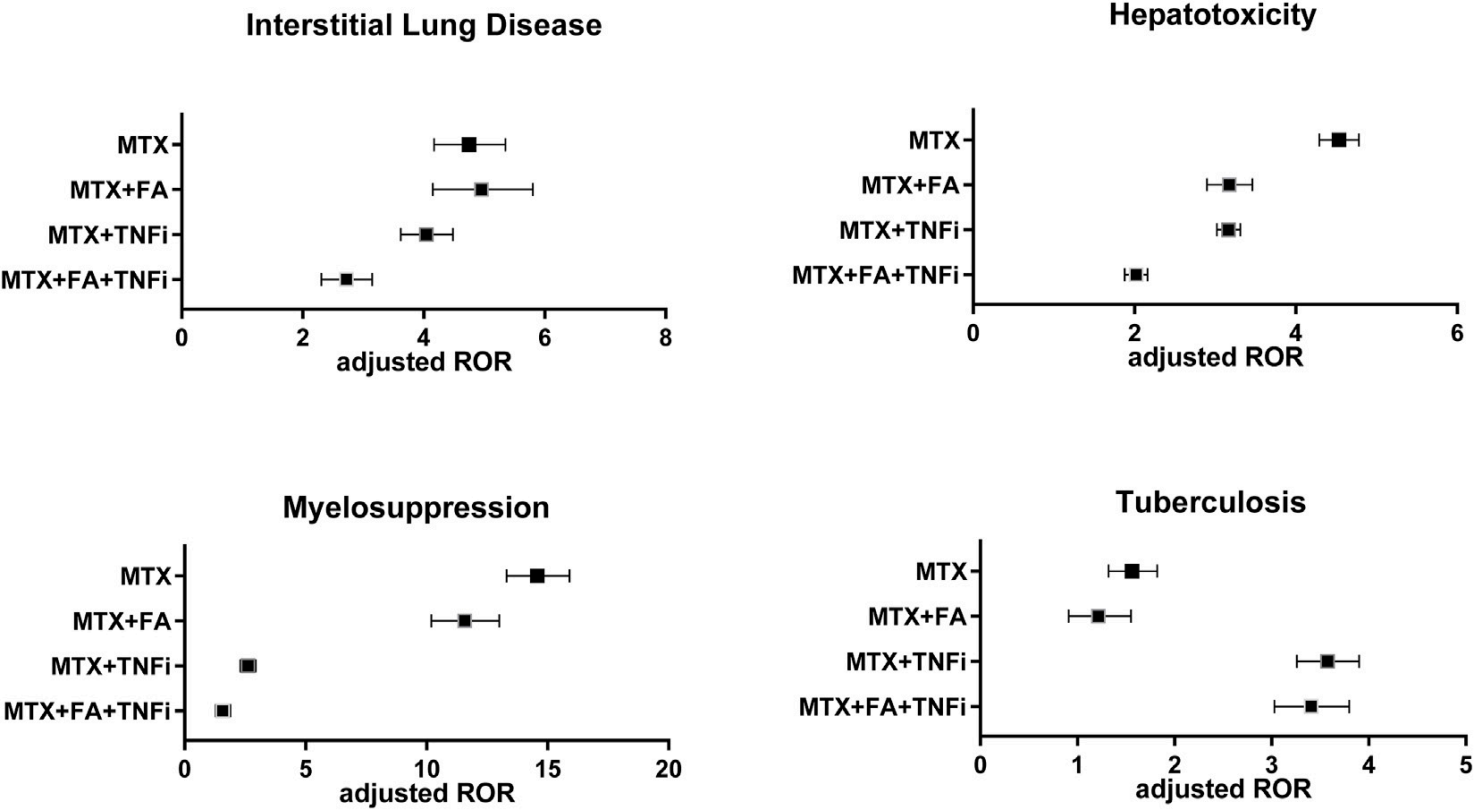
Adjusted reporting odds ratios (aRORs) (95% CI) for MTX-related adverse events (interstitial lung disease, hepatotoxicity, myelosuppression, and tuberculosis) calculated using the logistic regression analysis for each treatment pattern containing MTX (MTX, MTX + FA, MTX + TNFi, or MTX + FA + TNFi). CI, confidence interval; MTX, methotrexate; RORs, reporting odds ratios; FA, folic acid; TNFi, tumor necrosis factor inhibitor.

Finally, cRORs and aRORs for TB were examined. The aROR of the MTX group was 1.55 (1.32–1.82), whereas that of the MTX + FA group was 1.19 (0.91–1.55), which was not statistically significant (*p* = 0.206) and tended to be less than that of the MTX group. The MTX + TNFi group presented the highest aROR value [3.57 (3.26–3.9)], while the MTX + FA + TNFi group exhibited a comparable aROR value [3.39 (3.03–3.8)]. Figure 3 compares aRORs for four adverse events among the MTX treatment patterns.

## 4 Discussion

In the present study, we analyzed the impact of typical MTX-induced adverse effects, including ILD, hepatotoxicity, myelosuppression, and TB, on RORs considering concomitant medications using the FAERS database, one of the world’s largest spontaneous reporting databases. We calculated cRORs by performing a univariate analysis for all cases, and the relevant concomitant drugs (FA and TNFi) were incorporated into the explanatory variables in the multivariate analysis with RA cases. As it is regarded inappropriate to directly compare cROR values (Suzuki et al., 2015), we calculated aRORs for the MTX-related adverse events.

For multivariate analysis of drug combinations, logistic models incorporating interaction terms as covariants have been previously presented (van Puijenbroek et al., 2000), which were initially followed in our analysis. However, we detected VIF values ˃10 in each model, indicating the existence of multicollinearity in covariables. Therefore, we divided the reported cases of MTX treatment into four categories depending on MTX treatment patterns, establishing them as different covariables in our logistic model. Accordingly, we successfully confirmed that all VIF values were ˂1.4, and the statistical deviance was significantly small, which supports the validity of the logistic model.

ILD is a rare but critical side effect that can lead to severe outcomes. In the current study, a single PT was used to define an adverse event for ILD, which could distinguish adverse event reports from those of other types of lung disorders (e.g., pneumonia due to infection). A previous report using the FAERS database has shown that the cROR for ILD associated with MTX use was 3.25 (Matsui et al., 2015), which is comparable with the results of the present analysis. In the univariate analyses of cRORs for MTX and concomitant medications, the cRORs for ILD, hepatotoxicity, or myelosuppression tended to be significantly lower with the concomitant use of FA or TNFis (except for ILD with some TNFis). The cRORs for MTX and the concomitant use of classical DMARDs remained significantly high, suggesting that the concomitant use of FA or some TNFis exhibited distinct patterns of adverse event reports.

The univariate analysis to explore drugs that may influence MTX-related adverse events included all patient cases with and without RA. Besides, the data with cROR is not conclusive. Therefore, a multivariate analysis focusing on patients with RA was performed to assess the impact of FA and TNFis in those populations. Namely, RA cases aged ≤ 100 years were selected for multiple logistic analysis with age, sex, and MTX treatment patterns as explanatory variables. The aRORs for ILD revealed that male sex and older age were significantly associated with ILD reports, corroborating the results reported in a previous study (Fragoulis et al., 2019). We detected no apparent differences between the aRORs of the MTX + FA and MTX alone groups. This observation is consistent with previous studies reporting that FA does not reduce the ILD risk (Jakubovic et al., 2013). However, the MTX + TNFi group exhibited a lower aROR value than the MTX alone group, indicating the presence of fewer reports of ILD in the MTX + TNFi group. Although the underlying reason for this observation remains unclear, we propose that treatment with TNFi reduces the amount of MTX required to control disease activity. Conversely, some studies have reported that TNFi increases the potential risk of ILD (Nakashita et al., 2014; Huang et al., 2019; Taki et al., 2009); hence, further in-depth investigations are warranted.

The benefit of FA administration on hepatotoxicity, a dose-dependent side effect of MTX, is well established. FA has been shown to exert a preventive effect on MTX-induced abnormal elevation in serum transaminase levels (Shea et al., 2013). In the current multivariate analysis, MTX combined with FA afforded a lower aROR value than MTX alone. Furthermore, the MTX + FA + TNFi group presented a lower aROR value than the MTX + TNFi combination group, suggesting that FA administration affects MTX-induced hepatotoxicity, regardless of TNFi use.

MTX inhibits folate metabolism, which is necessary for DNA synthesis during cell proliferation and can thus suppress hematopoietic cell production. The side effects of myelosuppression may be severe and dose dependent. However, previous reports on the effects of FA administration have failed to establish a notable FA-mediated prophylactic effect on myelosuppression. Thus, evidence supporting FA-mediated prevention of neutropenia or impaired leukocyte production remains poorly established (Shea et al., 2013). Recently, FA was shown to prevent serious hematologic toxicity in MTX users with chronic kidney disease and RA in an analysis of the Japanese version of the Spontaneous Reporting System (JADER) (Mitsuboshi, 2021).

In our FAERS analysis, the aROR of myelosuppression was lower in groups administering FA than in those without FA administration. We also observed that there were fewer adverse myelosuppression events when MTX was combined with TNFi. In addition, FA administration was associated with fewer adverse event reports of myelosuppression, both with and without TNFi use. Further validation is required to determine whether the reduced aRORs for myelosuppression in the MTX + TNFi group, compared with the MTX alone group, or the MTX + FA + TNFi group, compared to the MTX + FA group, were related to the decreased MTX dose owing to TNFi administration.

With regard to MTX dosage, the distribution of RA patients in relation to the MTX dosage (maximum dose per week) was shown in Supplementary Figure S1. However, for the following reasons, we believe that there are limitations in presenting vetted results for MTX dose comparisons among treatment patterns using FAERS (i.e., due to heterogeneity of descriptions in FAERS on doses, units, cumulative doses, the existence of multiple doses in the same report ID, unknown timing of dosing, and presence of deviations that appear to be based on misstatements). Therefore, verification by other research methods is warranted.

Alveolar macrophages secrete TNFα into the physiological environment. As TNFα plays a vital role in defense mechanisms against infections, TNFis increase the risk of infection, including TB. In the current analysis, the combination of MTX + TNFi showed a marked increase in aRORs of TB compared to that of MTX alone, highlighting the increased risk associated with combining both drugs. FA administration minimally impacted aRORs for TB.

Compared with MTX or TNFi alone, combining MTX and TNFi reportedly affords high efficacy in disease control (Klareskog et al., 2004). Herein, adverse event reports such as ILD, hepatotoxicity, and myelosuppression tended to reduce following combination therapy with MTX and TNFi when compared with MTX alone; however, the ROR for TB increased with the two-drug combination, suggesting that the concomitant use of MTX and TNFi should be considered carefully.

It should be noted that FAERS is subject to various reporting biases, given that it uses spontaneously reported data. The true frequency of adverse events cannot be assessed (Noguchi et al., 2021). Therefore, despite the calculation of aRORs, the findings observed in the present study do not necessarily reflect an actual situation. We cannot rule out the influence of potential confounders that were not included in the logistic regression model (Urushihara, 2020). The current analysis did not consider data on dose, timing, route of administration, disease type, or degree of progression. Other drugs that may cause these adverse events could impact the study results. Hence, the objective of the present study was to estimate the relationship between the analysis of spontaneously reported data and previous clinical or epidemiological studies and propose a new hypothesis. Despite these limitations, an analysis based on large-scale real-world data has significant advantages. Further evaluation using different approaches is required to complement these findings.

## 5 Conclusion

To the best of our knowledge, this is the first report to analyze the effect of concomitant medications on various MTX-related adverse events reported in FAERS. We demonstrated differences in the aRORs for the four adverse events depending on the treatment patterns of MTX therapy. In addition, FA could reduce the aROR for dose-dependent adverse events of MTX, which supports the results of previous epidemiological studies demonstrating the benefits of FA administration. The present study, incorporating our proposed logistic models for drug combinations, revealed the usefulness of analyzing the FAERS database for evaluating adverse events and comparing various treatment patterns.

## Data Availability

Publicly available datasets were analyzed in this study. This data can be found here: https://www.fda.gov/drugs/questions-and-answers-fdas-adverse-event-reporting-system-faers/fda-adverse-event-reporting-system-faers-public-dashboard.

## References

[B1] Atzeni F. Boiardi L. Sallì S. Benucci M. Sarzi-Puttini P. (2013). Lung involvement and drug-induced lung disease in patients with rheumatoid arthritis. Expert Rev. Clin. Immunol. 9, 649–657. 10.1586/1744666X.2013.811173 23899235

[B2] Bate A. Evans S. J. W. (2009). Quantitative signal detection using spontaneous ADR reporting. Pharmacoepidemiol. Drug Saf. 18, 427–436. 10.1002/pds.1742 19358225

[B3] Fleischmann R. Tongbram V. van Vollenhoven R. Tang D. H. Chung J. Collier D. (2017). Systematic review and network meta-analysis of the efficacy and safety of tumour necrosis factor inhibitor-methotrexate combination therapy versus triple therapy in rheumatoid arthritis. RMD Open 3, e000371. 10.1136/rmdopen-2016-000371 28123782 PMC5237767

[B4] Fragoulis G. E. Nikiphorou E. Larsen J. Korsten P. Conway R. (2019). Methotrexate-associated pneumonitis and rheumatoid arthritis-interstitial lung disease: Current concepts for the diagnosis and treatment. Front. Med. 6, 238. 10.3389/fmed.2019.00238 PMC6819370 31709258

[B5] Gandhi P. K. Gentry W. M. Bottorff M. B. (2013). Dabigatran-dronedarone interaction in a spontaneous reporting system. J. Am. Pharm. Assoc. 53, 414–419. 10.1331/JAPhA.2013.12218 23892815

[B6] Greenberg J. D. Reed G. Kremer J. M. Tindall E. Kavanaugh A. Zheng C. (2010). Association of methotrexate and tumour necrosis factor antagonists with risk of infectious outcomes including opportunistic infections in the CORRONA registry. Ann. Rheum. Dis. 69, 380–386. 10.1136/ard.2008.089276 19359261 PMC2861900

[B7] Ibrahim A. Ahmed M. Conway R. Carey J. J. (2018). Risk of infection with methotrexate therapy in inflammatory diseases: A systematic review and meta-analysis. J. Clin. Med. 8, E15. 10.3390/jcm8010015 PMC6352130 30583473

[B8] Ikemura K. Hiramatsu S.-I. Shinogi Y. Nakatani Y. Tawara I. Iwamoto T. (2019). Concomitant febuxostat enhances methotrexate-induced hepatotoxicity by inhibiting breast cancer resistance protein. Sci. Rep. 9, 20359. 10.1038/s41598-019-56900-2 31889141 PMC6937279

[B9] Iyer S. V. Harpaz R. LePendu P. Bauer-Mehren A. Shah N. H. (2014). Mining clinical text for signals of adverse drug-drug interactions. J. Am. Med. Inf. Assoc. JAMIA 21, 353–362. 10.1136/amiajnl-2013-001612 PMC3932451 24158091

[B10] Jakubovic B. D. Donovan A. Webster P. M. Shear N. H. (2013). Methotrexate-induced pulmonary toxicity. Can. Respir. J. 20, 153–155. 10.1155/2013/527912 23762881 PMC3814259

[B11] Kanda Y. (2013). Investigation of the freely available easy-to-use software “EZR” for medical statistics. Bone Marrow Transpl. 48, 452–458. 10.1038/bmt.2012.244 PMC3590441 23208313

[B12] Klareskog L. van der Heijde D. de Jager J. P. Gough A. Kalden J. Malaise M. (2004). Therapeutic effect of the combination of etanercept and methotrexate compared with each treatment alone in patients with rheumatoid arthritis: Double-blind randomised controlled trial. Lancet lond. Engl. 363, 675–681. 10.1016/S0140-6736(04)15640-7 15001324

[B13] Matsui T. Umetsu R. Kato Y. Ueda N. Abe J. Nakayama Y. (2015). Adverse event signals of interstitial lung disease in the FDA adverse event reporting system (FAERS) database and the Japanese adverse drug event report (JADER) database. Iyakuhin Johogaku 17, 145–154. 10.11256/jjdi.17.145

[B14] Matsumoto K. Nakao S. Hasegawa S. Matsui T. Shimada K. Mukai R. (2020). Analysis of drug-induced interstitial lung disease using the Japanese Adverse Drug Event Report database. SAGE Open Med. 8, 2050312120918264. 10.1177/2050312120918264 32528682 PMC7262990

[B15] Mitsuboshi S. (2021). Risk of haematological events and preventive effect of folic acid in methotrexate users with chronic kidney disease and rheumatoid arthritis: Analysis of the Japanese Adverse Drug Event Report database. Br. J. Clin. Pharmacol. 87, 2286–2289. 10.1111/bcp.14641 33179261

[B16] Nagaoka K. Nagashima T. Asaoka N. Yamamoto H. Toda C. Kayanuma G. (2021). Striatal TRPV1 activation by acetaminophen ameliorates dopamine D2 receptor antagonist-induced orofacial dyskinesia. JCI Insight 6, 145632. 10.1172/jci.insight.145632 33857021 PMC8262333

[B17] Noguchi Y. Tachi T. Teramachi H. (2021). Detection algorithms and attentive points of safety signal using spontaneous reporting systems as a clinical data source. Brief. Bioinform. 22, bbab347. 10.1093/bib/bbab347 34453158

[B18] Oshima Y. Tanimoto T. Yuji K. Tojo A. (2018). EGFR-TKI-Associated interstitial pneumonitis in nivolumab-treated patients with non-small cell lung cancer. JAMA Oncol. 4, 1112–1115. 10.1001/jamaoncol.2017.4526 29327061 PMC5885195

[B19] Sakaeda T. Tamon A. Kadoyama K. Okuno Y. (2013). Data mining of the public version of the FDA adverse event reporting system. Int. J. Med. Sci. 10, 796–803. 10.7150/ijms.6048 23794943 PMC3689877

[B20] Shea B. Swinden M. V. Ghogomu E. T. Ortiz Z. Katchamart W. Rader T. (2014). Folic acid and folinic acid for reducing side effects in patients receiving methotrexate for rheumatoid arthritis. J. Rheumatol. 41, 1049–1060. 10.3899/jrheum.130738 24737913

[B21] Shea B. Swinden M. V. Tanjong Ghogomu E. Ortiz Z. Katchamart W. Rader T. (2013). Folic acid and folinic acid for reducing side effects in patients receiving methotrexate for rheumatoid arthritis. Cochrane Database Syst. Rev. 2013, CD000951. 10.1002/14651858.CD000951.pub2 23728635 PMC7046011

[B22] Suzuki Y. Suzuki H. Umetsu R. Uranishi H. Abe J. Nishibata Y. (2015). Analysis of the interaction between clopidogrel, aspirin, and proton pump inhibitors using the FDA adverse event reporting system database. Biol. Pharm. Bull. 38, 680–686. 10.1248/bpb.b14-00191 25947914

[B23] Taki H. Kawagishi Y. Shinoda K. Hounoki H. Ogawa R. Sugiyama E. (2009). Interstitial pneumonitis associated with infliximab therapy without methotrexate treatment. Rheumatol. Int. 30, 275–276. 10.1007/s00296-009-0931-6 19373466

[B24] Urushihara H. (2020). Basic dos and don’ts in applying signal detection methods to spontaneous reporting systems databases. Jpn. J. Drug Inf. 21, 135–141. 10.11256/jjdi.21.135

[B25] van Puijenbroek E. P. Egberts A. C. Heerdink E. R. Leufkens H. G. (2000). Detecting drug-drug interactions using a database for spontaneous adverse drug reactions: An example with diuretics and non-steroidal anti-inflammatory drugs. Eur. J. Clin. Pharmacol. 56, 733–738. 10.1007/s002280000215 11214785

[B26] Zhao S. Nishimura T. Chen Y. Azeloglu E. U. Gottesman O. Giannarelli C. (2013). Systems pharmacology of adverse event mitigation by drug combinations. Sci. Transl. Med. 5, 206ra140. 10.1126/scitranslmed.3006548 PMC3963511 24107779

