# Supplementary material for: Methotrexate-related adverse events and impact of concomitant treatment with folic acid and tumor necrosis factor-alpha inhibitors: An assessment using the FDA adverse event reporting system
Source: Front Pharmacol. 2023 Feb 22;14:1030832. doi: 10.3389/fphar.2023.1030832 (PMC9992735; doi:10.3389/fphar.2023.1030832)
Supplement: Supplementary file 1 [file DataSheet1.docx]

Supplementary Material

# Supplementary Data

## Supplelentary Table 1 Complete lists of the prefered terms useded for definition of adverse events.

| **PT code for Interstitial lung disease** |  |
| --- | --- |
| Ptcode | PT term |
| 10022611 | Interstitial lung disease |
| **PT codes for Hepatotoxicity** |  |
| Ptcode | PT term |
| 10000028 | 5'nucleotidase increased |
| 10000804 | Acute hepatic failure |
| 10001547 | Alanine aminotransferase abnormal |
| 10001551 | Alanine aminotransferase increased |
| 10001942 | Ammonia abnormal |
| 10001946 | Ammonia increased |
| 10003445 | Ascites |
| 10003477 | Aspartate aminotransferase abnormal |
| 10003481 | Aspartate aminotransferase increased |
| 10003547 | Asterixis |
| 10003827 | Autoimmune hepatitis |
| 10004269 | Benign hepatic neoplasm |
| 10004659 | Biliary cirrhosis |
| 10004664 | Biliary fibrosis |
| 10004685 | Bilirubin conjugated increased |
| 10004792 | Biopsy liver abnormal |
| 10005364 | Blood bilirubin increased |
| 10005370 | Blood bilirubin unconjugated increased |
| 10005429 | Blood cholinesterase abnormal |
| 10005430 | Blood cholinesterase decreased |
| 10005518 | Blood fibrinogen abnormal |
| 10005520 | Blood fibrinogen decreased |
| 10005818 | Blood thrombin abnormal |
| 10005820 | Blood thrombin decreased |
| 10005824 | Blood thromboplastin abnormal |
| 10005826 | Blood thromboplastin decreased |
| 10006408 | Bromosulphthalein test abnormal |
| 10008635 | Cholestasis |
| 10008909 | Chronic hepatitis |
| 10009736 | Coagulation factor decreased |
| 10009746 | Coagulation factor IX level decreased |
| 10009754 | Coagulation factor V level decreased |
| 10009761 | Coagulation factor VII level decreased |
| 10009775 | Coagulation factor X level decreased |
| 10010075 | Coma hepatic |
| 10010186 | Complications of transplanted liver |
| 10017688 | Gamma-glutamyltransferase abnormal |
| 10017693 | Gamma-glutamyltransferase increased |
| 10018704 | Granulomatous liver disease |
| 10018821 | Haemangioma of liver |
| 10019621 | Hepaplastin abnormal |
| 10019622 | Hepaplastin decreased |
| 10019629 | Hepatic adenoma |
| 10019637 | Hepatic atrophy |
| 10019641 | Hepatic cirrhosis |
| 10019646 | Hepatic cyst |
| 10019660 | Hepatic encephalopathy |
| 10019663 | Hepatic failure |
| 10019668 | Hepatic fibrosis |
| 10019670 | Hepatic function abnormal |
| 10019692 | Hepatic necrosis |
| 10019695 | Hepatic neoplasm |
| 10019705 | Hepatic pain |
| 10019708 | Hepatic steatosis |
| 10019717 | Hepatitis |
| 10019727 | Hepatitis acute |
| 10019754 | Hepatitis cholestatic |
| 10019755 | Hepatitis chronic active |
| 10019759 | Hepatitis chronic persistent |
| 10019772 | Hepatitis fulminant |
| 10019795 | Hepatitis toxic |
| 10019823 | Hepatoblastoma recurrent |
| 10019837 | Hepatocellular injury |
| 10019842 | Hepatomegaly |
| 10019845 | Hepatorenal failure |
| 10019846 | Hepatorenal syndrome |
| 10019847 | Hepatosplenomegaly |
| 10019851 | Hepatotoxicity |
| 10020575 | Hyperammonaemia |
| 10020578 | Hyperbilirubinaemia |
| 10020942 | Hypoalbuminaemia |
| 10020973 | Hypocoagulable state |
| 10021085 | Hypoprothrombinaemia |
| 10021209 | Icterus index increased |
| 10022592 | International normalised ratio abnormal |
| 10022595 | International normalised ratio increased |
| 10023025 | Ischaemic hepatitis |
| 10023126 | Jaundice |
| 10023129 | Jaundice cholestatic |
| 10023136 | Jaundice hepatocellular |
| 10023321 | Kayser-Fleischer ring |
| 10024275 | Leucine aminopeptidase increased |
| 10024670 | Liver disorder |
| 10024690 | Liver function test abnormal |
| 10024712 | Liver tenderness |
| 10024714 | Liver transplant |
| 10024715 | Liver transplant rejection |
| 10025129 | Lupoid hepatic cirrhosis |
| 10027761 | Mixed hepatocellular cholangiocarcinoma |
| 10030210 | Oesophageal varices haemorrhage |
| 10036200 | Portal hypertension |
| 10036204 | Portal shunt |
| 10037005 | Protein C decreased |
| 10037048 | Prothrombin level abnormal |
| 10037050 | Prothrombin level decreased |
| 10037057 | Prothrombin time abnormal |
| 10037063 | Prothrombin time prolonged |
| 10037068 | Prothrombin time ratio increased |
| 10039012 | Reye's syndrome |
| 10041519 | Spider naevus |
| 10041661 | Splenorenal shunt |
| 10045428 | Ultrasound liver abnormal |
| 10048245 | Yellow skin |
| 10048611 | Cholaemia |
| 10049483 | Glutamate dehydrogenase increased |
| 10049547 | Antithrombin III decreased |
| 10049631 | Oedema due to hepatic disease |
| 10050792 | Urine bilirubin increased |
| 10050842 | Liver carcinoma ruptured |
| 10050897 | Portal hypertensive gastropathy |
| 10051010 | Duodenal varices |
| 10051012 | Gastric varices |
| 10051015 | Radiation hepatitis |
| 10051081 | Nodular regenerative hyperplasia |
| 10051120 | Protein S decreased |
| 10051125 | Hypofibrinogenaemia |
| 10051319 | Thrombin time abnormal |
| 10051343 | Bile output decreased |
| 10051344 | Bile output abnormal |
| 10051390 | Thrombin time prolonged |
| 10051603 | Liver and pancreas transplant rejection |
| 10051736 | Protein S abnormal |
| 10051924 | Hypercholia |
| 10052274 | Hepatopulmonary syndrome |
| 10052279 | Renal and liver transplant |
| 10052285 | Focal nodular hyperplasia |
| 10052550 | Liver induration |
| 10052554 | Foetor hepaticus |
| 10052716 | Peritoneovenous shunt |
| 10053219 | Non-alcoholic steatohepatitis |
| 10053244 | Hepatocellular foamy cell syndrome |
| 10053973 | Hepatic cyst ruptured |
| 10054125 | Perihepatic discomfort |
| 10054885 | Hepatic haemangioma rupture |
| 10054889 | Transaminases increased |
| 10055110 | Hepatic cancer metastatic |
| 10056091 | Varices oesophageal |
| 10056536 | X-ray hepatobiliary abnormal |
| 10056956 | Subacute hepatic failure |
| 10057110 | Hepatic mass |
| 10057572 | Gastric varices haemorrhage |
| 10057573 | Chronic hepatic failure |
| 10058117 | Ocular icterus |
| 10058477 | Blood bilirubin abnormal |
| 10058517 | Hypothrombinaemia |
| 10059318 | Hepatic cancer stage I |
| 10059319 | Hepatic cancer stage II |
| 10059324 | Hepatic cancer stage III |
| 10059325 | Hepatic cancer stage IV |
| 10059570 | Blood alkaline phosphatase increased |
| 10059571 | Blood alkaline phosphatase abnormal |
| 10059766 | Haemorrhagic ascites |
| 10060794 | Hepatic enzyme decreased |
| 10060795 | Hepatic enzyme increased |
| 10061009 | Bilirubin excretion disorder |
| 10061203 | Hepatobiliary neoplasm |
| 10061770 | Coagulation factor IX level abnormal |
| 10061771 | Coagulation factor V level abnormal |
| 10061772 | Coagulation factor VII level abnormal |
| 10061774 | Coagulation factor X level abnormal |
| 10061918 | Prothrombin time ratio abnormal |
| 10061947 | Liver scan abnormal |
| 10061997 | Hepatectomy |
| 10061998 | Hepatic lesion |
| 10062000 | Hepatobiliary disease |
| 10062001 | Hepatoblastoma |
| 10062040 | Liver operation |
| 10062685 | Hepatic enzyme abnormal |
| 10062688 | Transaminases abnormal |
| 10063075 | Cryptogenic cirrhosis |
| 10064190 | Cholestatic pruritus |
| 10064558 | Total bile acids increased |
| 10064668 | Hepatic infiltration eosinophilic |
| 10064676 | Graft versus host disease in liver |
| 10064712 | Mitochondrial aspartate aminotransferase increased |
| 10064936 | Portal vein pressure increased |
| 10065274 | Hepatic calcification |
| 10066004 | Pneumobilia |
| 10066195 | Hepatobiliary scan abnormal |
| 10066244 | Hepatic sequestration |
| 10066263 | Acute graft versus host disease in liver |
| 10066599 | Hepatic encephalopathy prophylaxis |
| 10066758 | Mixed liver injury |
| 10066869 | Molar ratio of total branched-chain amino acid to tyrosine |
| 10067125 | Liver injury |
| 10067281 | Portopulmonary hypertension |
| 10067337 | Portal vein flow decreased |
| 10067338 | Retrograde portal vein flow |
| 10067365 | Hepatic hydrothorax |
| 10067388 | Hepatic angiosarcoma |
| 10067718 | Bilirubin conjugated abnormal |
| 10067737 | Lupus hepatitis |
| 10067796 | Haemorrhagic hepatic cyst |
| 10067823 | Splenic varices |
| 10067969 | Cholestatic liver injury |
| 10068237 | Hypertransaminasaemia |
| 10068287 | Child-Pugh-Turcotte score increased |
| 10068370 | Acquired protein S deficiency |
| 10068547 | Bacterascites |
| 10068662 | Splenic varices haemorrhage |
| 10068664 | Liver sarcoidosis |
| 10068821 | Periportal oedema |
| 10068923 | Portal hypertensive enteropathy |
| 10068924 | Anorectal varices |
| 10068925 | Anorectal varices haemorrhage |
| 10068997 | Hepatic artery flow decreased |
| 10069380 | Small-for-size liver syndrome |
| 10070479 | Urobilinogen urine increased |
| 10070953 | Reynold's syndrome |
| 10071198 | Allergic hepatitis |
| 10071265 | Diabetic hepatopathy |
| 10071502 | Intestinal varices |
| 10071634 | Deficiency of bile secretion |
| 10072160 | Chronic graft versus host disease in liver |
| 10072268 | Drug-induced liver injury |
| 10072284 | Varicose veins of abdominal wall |
| 10072319 | Gallbladder varices |
| 10072629 | Intrahepatic portal hepatic venous fistula |
| 10073069 | Hepatic cancer |
| 10073070 | Hepatic cancer recurrent |
| 10073071 | Hepatocellular carcinoma |
| 10073073 | Hepatobiliary cancer |
| 10073074 | Hepatobiliary cancer in situ |
| 10073209 | Portal vein dilatation |
| 10073215 | Peripancreatic varices |
| 10073979 | Portal vein cavernous transformation |
| 10074084 | Hepatic fibrosis marker abnormal |
| 10074150 | Biliary ascites |
| 10074151 | Parenteral nutrition associated liver disease |
| 10074352 | Liver iron concentration abnormal |
| 10074354 | Liver iron concentration increased |
| 10074413 | Hepatic fibrosis marker increased |
| 10074561 | Acquired antithrombin III deficiency |
| 10074726 | Portal fibrosis |
| 10074737 | Hyperfibrinolysis |
| 10075186 | Stomal varices |
| 10075331 | Portal tract inflammation |
| 10075895 | Liver palpable |
| 10076239 | Spontaneous intrahepatic portosystemic venous shunt |
| 10076254 | Hepatic hypertrophy |
| 10076331 | Steatohepatitis |
| 10076640 | Liver dialysis |
| 10077020 | Child-Pugh-Turcotte score abnormal |
| 10077215 | Hepatic steato-fibrosis |
| 10077259 | Non-cirrhotic portal hypertension |
| 10077281 | Splenorenal shunt procedure |
| 10077291 | Model for end stage liver disease score abnormal |
| 10077292 | Model for end stage liver disease score increased |
| 10077305 | Acute on chronic liver failure |
| 10077356 | Bilirubin urine present |
| 10077479 | Portal shunt procedure |
| 10077670 | Anti factor X activity abnormal |
| 10077671 | Anti factor X activity increased |
| 10077674 | Anti factor X activity decreased |
| 10077677 | Liver function test decreased |
| 10077692 | Liver function test increased |
| 10077861 | Cholangiosarcoma |
| 10077922 | Benign hepatobiliary neoplasm |
| 10078058 | Intestinal varices haemorrhage |
| 10078360 | Computerised tomogram liver abnormal |
| 10078962 | Immune-mediated hepatitis |
| 10079685 | Hepatic hamartoma |
| 10079686 | Hepatic lymphocytic infiltration |
| 10079889 | Hepatobiliary cyst |
| 10080429 | Primary biliary cholangitis |
| 10080824 | Glycocholic acid increased |
| 10082249 | Nonalcoholic fatty liver disease |
| 10082450 | Multivisceral transplantation |
|  |  |
| **PT codes for myelosuppression** |  |
| Ptcode | PT term |
| 10002967 | Aplastic anaemia |
| 10003506 | Aspiration bone marrow abnormal |
| 10004738 | Biopsy bone marrow abnormal |
| 10017413 | Full blood count decreased |
| 10028533 | Myelodysplastic syndrome |
| 10028537 | Myelofibrosis |
| 10028561 | Myeloid metaplasia |
| 10033661 | Pancytopenia |
| 10050026 | Panmyelopathy |
| 10053138 | Congenital aplastic anaemia |
| 10053213 | Febrile bone marrow aplasia |
| 10053504 | Scan bone marrow abnormal |
| 10057528 | Bone marrow myelogram abnormal |
| 10058822 | Bone marrow necrosis |
| 10058956 | Bicytopenia |
| 10061188 | Haematotoxicity |
| 10061590 | Blood disorder |
| 10061729 | Bone marrow disorder |
| 10064198 | Blood count abnormal |
| 10065553 | Bone marrow failure |
| 10066274 | Cytopenia |
| 10067387 | Myelodysplastic syndrome transformation |
| 10071576 | Autoimmune aplastic anaemia |
| 10075173 | Bone marrow infiltration |
| 10077161 | Primary myelofibrosis |
| 10078097 | Gelatinous transformation of the bone marrow |
|  |  |
| **PT codes for tuberculosis** |  |
| Ptcode | PT term |
| 10001358 | Adrenal gland tuberculosis |
| 10008779 | Choroid tubercles |
| 10010657 | Congenital tuberculosis |
| 10011684 | Cutaneous tuberculosis |
| 10013453 | Disseminated tuberculosis |
| 10014027 | Ear tuberculosis |
| 10015004 | Epididymitis tuberculous |
| 10025183 | Lymph node tuberculosis |
| 10027259 | Meningitis tuberculous |
| 10030200 | Oesophageal tuberculosis |
| 10037440 | Pulmonary tuberculosis |
| 10038534 | Renal tuberculosis |
| 10039463 | Salpingitis tuberculous |
| 10041640 | Spleen tuberculosis |
| 10043774 | Thyroid tuberculosis |
| 10044725 | Tuberculid |
| 10044729 | Tuberculoid leprosy |
| 10044755 | Tuberculosis |
| 10044758 | Tuberculosis bladder |
| 10044819 | Tuberculosis of eye |
| 10044828 | Tuberculosis of genitourinary system |
| 10044846 | Tuberculosis of intrathoracic lymph nodes |
| 10044965 | Tuberculosis of peripheral lymph nodes |
| 10045026 | Tuberculosis ureter |
| 10045072 | Tuberculous laryngitis |
| 10045104 | Tuberculous pleurisy |
| 10052883 | Tuberculoma of central nervous system |
| 10052884 | Tuberculous abscess central nervous system |
| 10053583 | Peritoneal tuberculosis |
| 10055069 | Pericarditis tuberculous |
| 10056367 | Joint tuberculosis |
| 10056377 | Bone tuberculosis |
| 10058120 | Tuberculosis liver |
| 10059161 | Tuberculous tenosynovitis |
| 10061150 | Female genital tract tuberculosis |
| 10061234 | Male genital tract tuberculosis |
| 10061390 | Tuberculosis gastrointestinal |
| 10061391 | Tuberculosis of central nervous system |
| 10063050 | False positive tuberculosis test |
| 10064445 | Extrapulmonary tuberculosis |
| 10064743 | Prostatitis tuberculous |
| 10065048 | Latent tuberculosis |
| 10066927 | Pulmonary tuberculoma |
| 10070325 | Mycobacterium tuberculosis complex test positive |
| 10070471 | Mycobacterium tuberculosis complex test negative |
| 10070472 | Mycobacterium tuberculosis complex test |
| 10072797 | Immune reconstitution inflammatory syndrome associated tuberculosis |
| 10075268 | Intestinal tuberculosis |
| 10076879 | Oral tuberculosis |

## Supplelentary Table 2 Crude reporting odds ratios of MTX-related adverse events among cases where MTX and concomitant Drug B was used.

| **ILD in cases of MTX use** | MTX with DrugB | | | MTX without DrugB | | |  |  |
| --- | --- | --- | --- | --- | --- | --- | --- | --- |
|  | cases | noncases |  | cases | noncases |  | crude ROR | Χ2 |
| DrugB | n11 | n12 | % | n21 | n22 | % |  |  |
| adalimumab | 466 | 70,779 | 0.65 | 1,900 | 218,691 | 0.86 | 0.76 (0.68-0.84) | 28.76 |
| infliximab | 319 | 31,735 | 1.00 | 2,047 | 257,735 | 0.79 | 1.27 (1.12-1.42) | 15.24 |
| etanercept | 622 | 82,137 | 0.75 | 1,744 | 207,333 | 0.83 | 0.9 (0.82-0.99) | 5.03 |
| certolizumab | 148 | 9,400 | 1.55 | 2,218 | 280,070 | 0.79 | 1.99 (1.68-2.35) | 67.1 |
| golimumab | 129 | 9,082 | 1.40 | 2,237 | 280,388 | 0.79 | 1.78 (1.49-2.13) | 41.14 |
| folic acid | 556 | 74,419 | 0.74 | 1,810 | 215,051 | 0.83 | 0.89 (0.81-0.98) | 6 |
| actarit | 15 | 70 | 17.65 | 2,351 | 289,400 | 0.81 | 26.38 (15.08-46.14) | 299.71 |
| lobenzarit | 2 | 8 | 20 | 2,364 | 289,462 | 0.81 | 30.61 (6.5-144.23) | 45.79 |
| auranofin | 2 | 96 | 2 | 2,364 | 289,374 | 0.81 | 2.55 (0.63-10.35) | 1.84 |
| sodium aurothiomalate | 26 | 809 | 3 | 2,340 | 288,661 | 0.80 | 3.96 (2.68-5.87) | 55.23 |
| mizoribine | 17 | 181 | 8.59 | 2,349 | 289,289 | 0.81 | 11.57 (7.02-19.05) | 148.95 |
| sulfasalazine | 344 | 16,602 | 2.03 | 2,022 | 272,868 | 0.74 | 2.8 (2.49-3.14) | 332.58 |
| bucillamine | 48 | 640 | 6.98 | 2,318 | 288,830 | 0.80 | 9.35 (6.95-12.57) | 326.05 |
| leflunomide | 377 | 21,586 | 1.72 | 1,989 | 267,884 | 0.74 | 2.35 (2.11-2.63) | 242.32 |
| tacrolimus | 146 | 4,434 | 3.19 | 2,220 | 285,036 | 0.77 | 4.23 (3.57-5.01) | 326.94 |
| iguratimod | 9 | 140 | 6.04 | 2,357 | 289,330 | 0.81 | 7.89 (4.02-15.5) | 50.7 |
| hydroxychloroquine | 435 | 32,157 | 1.33 | 1,931 | 257,313 | 0.74 | 1.8 (1.62-2) | 125.25 |
| abatacept | 336 | 17,699 | 1.86 | 2,030 | 271,771 | 0.74 | 2.54 (2.26-2.86) | 264.71 |
| tocilizumab | 323 | 15,449 | 2.05 | 2,043 | 274,021 | 0.74 | 2.8 (2.49-3.16) | 317.36 |
| tofacitinib | 220 | 17,864 | 1.22 | 2,146 | 271,606 | 0.78 | 1.56 (1.36-1.79) | 39.48 |
| baricitinib | 2 | 106 | 1.85 | 2,364 | 289,364 | 0.81 | 2.31 (0.57-9.36) | 1.46 |
|  |  |  |  |  |  |  |  |  |
|  |  |  |  |  |  |  |  |  |
| **Hepatotoxicity in cases of MTX use** | MTX with DrugB | | | MTX without DrugB | | |  |  |
|  | cases | noncases |  | cases | noncases |  | crude ROR | Χ2 |
| DrugB | n11 | n12 | % | n21 | n22 | % |  |  |
| adalimumab | 3,956 | 67,289 | 5.55 | 16,155 | 204,436 | 7.32 | 0.74 (0.72-0.77) | 263.19 |
| infliximab | 2,309 | 29,745 | 7.20 | 17,802 | 241,980 | 6.85 | 1.06 (1.01-1.1) | 5.47 |
| etanercept | 4,696 | 78,063 | 5.67 | 15,415 | 193,662 | 7.37 | 0.76 (0.73-0.78) | 266.6 |
| certolizumab | 589 | 8,959 | 6.17 | 19,522 | 262,766 | 6.92 | 0.88 (0.81-0.96) | 8.03 |
| golimumab | 575 | 8,636 | 6.24 | 19,536 | 263,089 | 6.91 | 0.9 (0.82-0.98) | 6.24 |
| folic acid | 3,174 | 71,801 | 4.23 | 16,937 | 199,924 | 7.81 | 0.52 (0.5-0.54) | 1110.78 |
| actarit | 19 | 66 | 22.35 | 20,092 | 271,659 | 6.89 | 3.89 (2.34-6.49) | 31.68 |
| lobenzarit | 4 | 6 | 40 | 20,107 | 271,719 | 6.89 | 9.01 (2.54-31.93) | 17.09 |
| auranofin | 14 | 84 | 14 | 20,097 | 271,641 | 6.89 | 2.25 (1.28-3.97) | 8.35 |
| sodium aurothiomalate | 158 | 677 | 19 | 19,953 | 271,048 | 6.86 | 3.17 (2.66-3.77) | 188.91 |
| mizoribine | 14 | 184 | 7.07 | 20,097 | 271,541 | 6.89 | 1.03 (0.6-1.77) | 0.01 |
| sulfasalazine | 1,727 | 15,219 | 10.19 | 18,384 | 256,506 | 6.69 | 1.58 (1.5-1.67) | 305.34 |
| bucillamine | 105 | 583 | 15.26 | 20,006 | 271,142 | 6.87 | 2.44 (1.98-3.01) | 75.3 |
| leflunomide | 2,864 | 19,099 | 13.04 | 17,247 | 252,626 | 6.39 | 2.2 (2.11-2.29) | 1399.53 |
| tacrolimus | 565 | 4,015 | 12.34 | 19,546 | 267,710 | 6.80 | 1.93 (1.76-2.11) | 215.01 |
| iguratimod | 14 | 135 | 9.40 | 20,097 | 271,590 | 6.89 | 1.4 (0.81-2.43) | 1.46 |
| hydroxychloroquine | 2,324 | 30,268 | 7.13 | 17,787 | 241,457 | 6.86 | 1.04 (1-1.09) | 3.28 |
| abatacept | 1,673 | 16,362 | 9.28 | 18,438 | 255,363 | 6.73 | 1.42 (1.34-1.49) | 170.45 |
| tocilizumab | 2,005 | 13,767 | 12.71 | 18,106 | 257,958 | 6.56 | 2.07 (1.98-2.18) | 880.55 |
| tofacitinib | 1,470 | 16,614 | 8.13 | 18,641 | 255,111 | 6.81 | 1.21 (1.15-1.28) | 46.02 |
| baricitinib | 13 | 95 | 12.04 | 20,098 | 271,630 | 6.89 | 1.85 (1.04-3.3) | 4.46 |
|  |  |  |  |  |  |  |  |  |
| **Myelosuppression in cases of MTX use** | MTX with DrugB | | | MTX without DrugB | | |  |  |
|  | cases | noncases |  | cases | noncases |  | crude ROR | Χ2 |
| DrugB | n11 | n12 | % | n21 | n22 | % |  |  |
| adalimumab | 350 | 70,895 | 0.49 | 7,972 | 212,619 | 3.61 | 0.13 (0.12-0.15) | 1895.53 |
| infliximab | 265 | 31,789 | 0.83 | 8,057 | 251,725 | 3.10 | 0.26 (0.23-0.29) | 532.95 |
| etanercept | 499 | 82,260 | 0.60 | 7,823 | 201,254 | 3.74 | 0.16 (0.14-0.17) | 2108.46 |
| certolizumab | 41 | 9,507 | 0.43 | 8,281 | 274,007 | 2.93 | 0.14 (0.1-0.19) | 209.05 |
| golimumab | 53 | 9,158 | 0.58 | 8,269 | 274,356 | 2.93 | 0.19 (0.15-0.25) | 177.88 |
| folic acid | 1,146 | 73,829 | 1.53 | 7,176 | 209,685 | 3.31 | 0.45 (0.43-0.48) | 637.57 |
| actarit | 11 | 74 | 12.94 | 8,311 | 283,440 | 2.85 | 5.07 (2.69-9.55) | 31.24 |
| lobenzarit | 0 | 10 | 0 | 8,322 | 283,504 | 2.85 | - | - |
| auranofin | 6 | 92 | 6 | 8,316 | 283,422 | 2.85 | 2.22 (0.97-5.08) | 3.79 |
| sodium aurothiomalate | 21 | 814 | 3 | 8,301 | 282,700 | 2.85 | 0.88 (0.57-1.36) | 0.34 |
| mizoribine | 10 | 188 | 5.05 | 8,312 | 283,326 | 2.85 | 1.81 (0.96-3.43) | 3.46 |
| sulfasalazine | 266 | 16,680 | 1.57 | 8,056 | 266,834 | 2.93 | 0.53 (0.47-0.6) | 106.72 |
| bucillamine | 52 | 636 | 7.56 | 8,270 | 282,878 | 2.84 | 2.8 (2.11-3.71) | 55.14 |
| leflunomide | 403 | 21,560 | 1.83 | 7,919 | 261,954 | 2.93 | 0.62 (0.56-0.68) | 88.62 |
| tacrolimus | 179 | 4,401 | 3.91 | 8,143 | 279,113 | 2.83 | 1.39 (1.2-1.62) | 18.75 |
| iguratimod | 9 | 140 | 6.04 | 8,313 | 283,374 | 2.85 | 2.19 (1.12-4.3) | 5.47 |
| hydroxychloroquine | 358 | 32,234 | 1.10 | 7,964 | 251,280 | 3.07 | 0.35 (0.32-0.39) | 407.07 |
| abatacept | 108 | 17,927 | 0.60 | 8,214 | 265,587 | 3.00 | 0.19 (0.16-0.24) | 352.15 |
| tocilizumab | 164 | 15,608 | 1.04 | 8,158 | 267,906 | 2.96 | 0.35 (0.3-0.4) | 197.56 |
| tofacitinib | 106 | 17,978 | 0.59 | 8,216 | 265,536 | 3.00 | 0.19 (0.16-0.23) | 357.16 |
| baricitinib | 1 | 107 | 0.93 | 8,321 | 283,407 | 2.85 | 0.32 (0.04-2.28) | 1.45 |
|  |  |  |  |  |  |  |  |  |
| **Tuberculosis in cases of MTX use** | MTX with DrugB | | | MTX without DrugB | | |  |  |
|  | cases | noncases |  | cases | noncases |  | crude ROR | Χ2 |
| DrugB | n11 | n12 | % | n21 | n22 | % |  |  |
| adalimumab | 597 | 70,648 | 0.84 | 2,224 | 218,367 | 1.01 | 0.83 (0.76-0.91) | 16.3 |
| infliximab | 1,154 | 30,900 | 3.60 | 1,667 | 258,115 | 0.64 | 5.78 (5.36-6.24) | 2608.82 |
| etanercept | 468 | 82,291 | 0.57 | 2,353 | 206,724 | 1.13 | 0.5 (0.45-0.55) | 194.18 |
| certolizumab | 187 | 9,361 | 1.96 | 2,634 | 279,654 | 0.93 | 2.12 (1.83-2.46) | 101.45 |
| golimumab | 122 | 9,089 | 1.32 | 2,699 | 279,926 | 0.95 | 1.39 (1.16-1.67) | 12.72 |
| folic acid | 780 | 74,195 | 1.04 | 2,041 | 214,820 | 0.94 | 1.11 (1.02-1.2) | 5.73 |
| actarit | 3 | 82 | 3.53 | 2,818 | 288,933 | 0.97 | 3.75 (1.18-11.88) | 5.83 |
| lobenzarit | 1 | 9 | 10 | 2,820 | 289,006 | 0.97 | 11.39 (1.44-89.91) | 8.52 |
| auranofin | 6 | 92 | 6 | 2,815 | 288,923 | 0.96 | 6.69 (2.93-15.3) | 27.22 |
| sodium aurothiomalate | 14 | 821 | 2 | 2,807 | 288,194 | 0.96 | 1.75 (1.03-2.97) | 4.41 |
| mizoribine | 7 | 191 | 3.54 | 2,814 | 288,824 | 0.96 | 3.76 (1.77-8) | 13.66 |
| sulfasalazine | 247 | 16,699 | 1.46 | 2,574 | 272,316 | 0.94 | 1.56 (1.37-1.78) | 45.29 |
| bucillamine | 31 | 657 | 4.51 | 2,790 | 288,358 | 0.96 | 4.88 (3.4-7.01) | 90.23 |
| leflunomide | 227 | 21,736 | 1.03 | 2,594 | 267,279 | 0.96 | 1.08 (0.94-1.23) | 1.11 |
| tacrolimus | 34 | 4,546 | 0.74 | 2,787 | 284,469 | 0.97 | 0.76 (0.54-1.07) | 2.44 |
| iguratimod | 2 | 147 | 1.34 | 2,819 | 288,868 | 0.97 | 1.39 (0.35-5.63) | 0.22 |
| hydroxychloroquine | 243 | 32,349 | 0.75 | 2,578 | 256,666 | 0.99 | 0.75 (0.66-0.85) | 18.73 |
| abatacept | 73 | 17,962 | 0.40 | 2,748 | 271,053 | 1.00 | 0.4 (0.32-0.51) | 63.39 |
| tocilizumab | 59 | 15,713 | 0.37 | 2,762 | 273,302 | 1.00 | 0.37 (0.29-0.48) | 61.16 |
| tofacitinib | 127 | 17,957 | 0.70 | 2,694 | 271,058 | 0.98 | 0.71 (0.6-0.85) | 14.07 |
| baricitinib | 2 | 106 | 1.85 | 2,819 | 288,909 | 0.97 | 1.93 (0.48-7.84) | 0.88 |

## Supplementary Figure 1 Distribution of the number of rheumatoid arthritis cases in relation to the maximal weekly dose of methotrexate (mg/week) listed in FAERS.
